# Investigation of Inherited Chromosomally Integrated Human Herpesvirus-6A+ and -6B+ in a Patient with Ulipristal Acetate-Induced Fulminant Hepatic Failure

**DOI:** 10.3390/v14010062

**Published:** 2021-12-30

**Authors:** Laure Izquierdo, Clémence M. Canivet, Eleonora De Martin, Teresa M. Antonini, Anne-Marie Roque-Afonso, Audrey Coilly, Claire Deback

**Affiliations:** 1 Laboratoire de Virologie, Institut National de la Santé et de la Recherche Médicale Unité 1193 AP-HP, Hôpitaux Universitaires Paris Saclay, Hôpital Paul-Brousse, 94800 Villejuif, France; laure.izquierdo@gmail.com (L.I.); anne-marie.roque@aphp.fr (A.-M.R.-A.); 2 Laboratoire HIFIH, UPRES EA3859, SFR 4208, Service d’Hépato-Gastroentérologie et Oncologie Digestive, Centre Hospitalier Universitaire d’Angers, Université d’Angers, 49000 Angers, France; clem.canivet@gmail.com; 3 Centre Hépato-Biliaire, AP-HP, Institut National de la Santé et de la Recherche Médicale Unité 1193, Hôpitaux Universitaires Paris Saclay, Hôpital Paul-Brousse, 94800 Villejuif, France; eleonora.demartin@aphp.fr (E.D.M.); teresa.antonini05@aphp.fr (T.M.A.); audrey.coilly@aphp.fr (A.C.); 4 Laboratoire de Virologie, AP-HP, Hôpitaux Universitaires Paris Saclay, Hôpital Paul-Brousse, 94800 Villejuif, France; 5 Inserm U996, Inflammation, Microbiome and Immunosurveillance, Université Paris-Saclay, 92140 Clamart, France

**Keywords:** human herpesvirus 6, HHV-6A, HHV-6B, iciHHV-6, ciHHV-6, integration, viral hepatitis, drug-induced liver injury, transplantation

## Abstract

Inherited chromosomally integrated (ici) human herpes virus 6 (HHV-6) is estimated to occur in 0.6–2.7% of people worldwide. HHV-6 comprises two distinct species: HHV-6A and HHV-6B. Both HHV-6A and HHV-6B integration have been reported. Several drugs are capable of activating iciHHV-6 in tissues, the consequences of which are poorly understood. We report herein a case of a woman with iciHHV-6A+ and iciHHV-6B+, who developed ulipristal acetate (a selective progesterone receptor modulator)-induced fulminant hepatic failure that required liver transplantation. We confirmed the presence of ~one copy per cell of both HHV-6A and HHV-6B DNA in her hair follicles using multiplex HHV-6A/B real-time PCR and demonstrated the Mendelian inheritance of both iciHHV-6A and iciHHV-6B in her family members over three generations. Because of the rarity of this presentation, we discuss herein the possible links between reactivated HHV-6 from iciHHV-6A and/or iciHHV-6B and adverse drug reactions, suggesting that iciHHV-6 could be screened before the introduction of any hepatotoxic drugs to exclude HHV-6 active disease or combined idiosyncratic drug-induced liver injury in these patients.

## 1. Introduction

Human herpesvirus 6 (HHV-6) in the genus *Roseolovirus*, subfamily *Betaherpesvirinae*, is divided into two distinct species, HHV-6A and HHV-6B [1]. Although the full genomic identity between the two species is 90%, HHV-6A and HHV-6B exhibit specific epidemiological, biological, and immunological properties that have not been extensively clarified. HHV-6 is a ubiquitous virus, and more than 95% of humans after the age of three are seropositive for HHV-6. The major species prevalent in the USA, Europe, and Japan is HHV-6B [2]. Although epidemiology reports are limited, asymptomatic HHV-6A infections are acquired later in life in these areas, and some epidemiological studies have recorded HHV-6A in children from sub-Saharan Africa and the USA at both common and low prevalence [3,4].

Both HHV-6A and HHV-6B primary infections are asymptomatic or are associated with a benign skin rash in children, causing exanthema subitum, also known as roseola or sixth disease [3,5]. Severe diseases, such as acute hepatitis, including acute liver failure, gastroenteritis, colitis, or myocarditis, are rarely attributed to HHV-6 primary infection in immunocompetent individuals [6,7]. Both HHV-6A and HHV-6B establish lifelong latency in different cell types, including CD4-positive T lymphocytes, monocytes, and other epithelial, fibroblastic, and neuronal cells, and may reactivate, causing serious complications, particularly in immunocompromised patients. Indeed, reactivations can lead to hepatitis, encephalitis, pneumonitis, and bone marrow suppression in hematopoietic stem cell transplant (HSCT) or solid organ transplant (SOT) recipients [6,8]. However, HHV-6A and HHV-6B have important biological and immunological discrepancies. Notably, HHV-6A is more prevalent in neurological diseases [2] and, although the two species exert common effects on the immune system, they affect differently both cell-mediated and humoral immune responses, in addition to direct cytopathic effects, via specific cytokine modulation and expression of viral chemokines and chemokine receptors [9].

HHV-6A/B have a double-stranded DNA genome flanked by an array of hexanucleotide telomeric repeats (TTAGGG)n that are identical to human telomere sequences [10]. HHV-6 can integrate into human chromosomes, frequently targeting subtelomeric or telomeric junctions. Chromosomally integrated HHV-6 (ciHHV-6) is estimated to occur in 0.6–2.7% of people worldwide [11]. The HHV-6 genome is covalently integrated into the telomeres of host somatic or germinal cell chromosomes [11,12]. When HHV-6 DNA integrates into germ cells, inherited chromosomally integrated HHV-6 (iciHHV-6) can segregate in a Mendelian manner, with a 50% chance of being passed to the offspring [13,14,15,16]. iciHHV-6 is present in each nucleated cell of the body, with at least one integrated copy of HHV-6 per genome. Thus, individuals carrying iciHHV-6 are characterized by a constant high viral load >5.5 log_10_ copies/mL of whole blood and ≥1 copy/cell in hair follicles, which is currently used to demonstrate that the virus is not restricted to blood cells. Moreover, any persistence of a high HHV-6 load in blood despite antivirals such as ganciclovir or foscarnet that efficiently inhibit progeny virus production must prompt the exploration of iciHHV-6 in patients in order to guide medical care and avoid unusual or toxic antiviral therapy [17,18].

To date, either iciHHV-6A or HHV-6B has been reported [16]. Herein, we report for the first time a case of both iciHHV-6A+ and iciHHV-6B+ in a woman suffering from fulminant hepatic failure, previous given the wrong diagnosis of natural HHV-6A and/or HHV-6B reactivation. We confirmed the presence and vertical transmission of both iciHHV-6A and iciHHV-6B DNA in her family members over three generations.

## 2. Case Description

**Figure 1:**
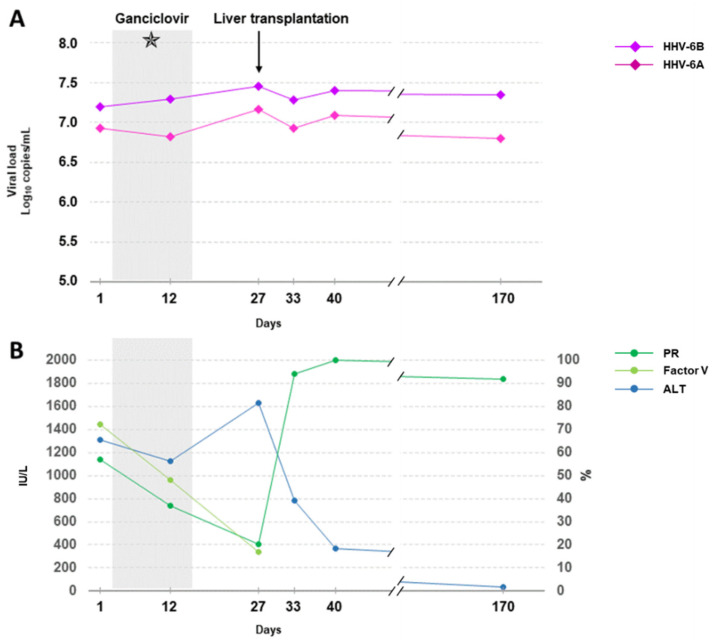
Kinetics of biomarkers and samples. (**A**) Loads of HHV-6A and -6B, expressed as log_10_ copies/mL of whole blood, before and after liver transplantation. The gray zone corresponds to two weeks of ganciclovir treatment. Hair follicle analysis for iciHHV-6A and/or iciHHV-6B (✯) was performed on the seventh day of the treatment; (**B**) ALT (IU/L), PR (%) and factor V (%) kinetics during the follow-up period. The gray zone corresponds to 2 weeks of ganciclovir treatment. ALT, alanine transaminase; PR, prothrombin ratio.

A 45-year-old woman was admitted to the emergency department because of elevated liver function tests in the context of mucocutaneous jaundice. The patient stated that she was not taking any toxic compounds or drinking alcohol. The patient had been treated with ulipristal acetate (Esmya^®^, Laboratoire Gedeon Richter, Paris, France) for 25 days for uterine fibroids. Since starting the therapy, she complained of asthenia, nausea and vomiting, cutaneous itching, and dark urine. Liver function tests revealed elevated levels of alanine aminotransferase (ALT) 1312 IU/L (normal range: 6 to 25 IU/L), elevated serum total bilirubin 330 µmol/L (normal <17 µmol/L), a low prothrombin ratio (PR) 57% (normal: >70%), and factor V 72% (normal: >60%). Liver biopsy from her native liver confirmed subacute hepatitis associated with lobular and periportal necrosis, involving 20% of the parenchyma and marked lymphocytic infiltrate. Repeated etiological evaluation included negative or normal serology for human immunodeficiency virus; human T cell leukemia virus type 1; hepatitis viruses A, B, C and E; cytomegalovirus (CMV) and Epstein–Barr virus (EBV). Quantitative real-time PCR was negative in blood for CMV, EBV, human herpes simplex virus type 1 and type 2, adenovirus, and hepatitis E virus. Conversely, HHV-6 DNAemia was positively detected with viral loads of 6.9 and 7.2 log_10_ copies/mL of whole blood for HHV-6A and -6B, respectively (Figure 1A). Briefly, DNA was purified from whole blood by using the VirusBlood 200 protocol on the Qiasymphony SP/AS instruments (Qiagen, Courtaboeuf, France) and the HHV-6A/-6B multiplex quantitative real-time PCR performed by using the artus^®^HHV-6 RG PCR Kit on the Rotor-Gene Q 5plex HRM System (Qiagen, Courtaboeuf, France), according to the manufacturer’s instructions.

Faced with possible subacute fulminant HHV-6A and/or HHV-6B-induced hepatitis, intravenous ganciclovir therapy was started at a dose of 5 mg per kilogram of body weight twice daily for 14 days (Figure 1). Due to rapid liver failure with an ALT level increase to 1600 IU/L (normal range: 6 to 25 IU/L), both prothrombin ratio (PR) < 25% (normal: >70%) and factor V decreases, and confirmed hepatic encephalopathy, the patient received a liver transplant at Day 27 (Figure 1). Initial immunosuppressive therapy included corticosteroid, mycophenolate mofetil and tacrolimus. Posttransplantation management was straightforward. Her ALT level gradually normalized, and PR rapidly improved (Figure 1B). Overall, a final diagnosis of fulminant drug-induced hepatitis due to ulipristal acetate (Esmya^®^, Laboratoire Gedeon Richter, Paris, France) was made.

## 3. Results

This case report illustrates the wrongful etiological diagnosis of subacute hepatitis in the context of iciHHV-6. In our patient, both HHV-6A (i.e., 6.9 log_10_ copies/mL) and HHV-6B (i.e., high loads of 7.2 log_10_ copies/mL) suggested a startling but possible ciHHV-6A+ and ciHHV-6B+ context, but we addressed the challenge of not ignoring the possibility of ongoing active HHV-6A and/or HHV-6B infections in our patient suffering from fulminant hepatic failure. The use of quantitative real-time PCR that distinguishes between HHV-6 species using fluorescent probes spectrally specific for HHV-6A DNA labeled with the fluorophore FAM™ and for HHV-6B DNA labeled with the fluorophore Cy^®^5, combined with the use of specific positive and negative controls, permitted us to eliminate any technical error or any cross-reaction between HHV-6A and HHV-6B primers and probes.

**Figure 2:**
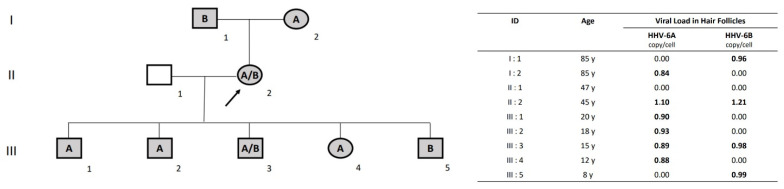
(**A**) Genealogical tree of the family harboring iciHHV-6A and/or iciHHV-6B. The arrow indicates the consultant patient (II:2) suffering from fulminant hepatic failure. (**B**) iciHHV-6A and/or iciHHV-6B viral loads determined in hair follicles and expressed in copy number per cell are indicated for each family member. ID, identification; y, year.

iciHHV-6A+ and iciHHV-6B+ cells were rapidly confirmed at Day 7 (Figure 1) by the evidence of both HHV-6A and HHV-6B DNA in hair follicles. Briefly, DNA was purified from three hair follicles using the NucliSENS ^®^ easyMAG^TM^ (Marcy l’Etoile) system, according to the manufacturer’s instruction. Both HHV-6A/-6B and human cell DNAs were quantified in parallel reactions by using the artus^®^HHV-6 RG PCR Kit on the Rotor-Gene Q 5plex HRM System (Qiagen, Courtaboeuf, France) as described above, and the CELL Control R-GENE^®^ kit (bioMérieux, Marcy l’Etoile, France), respectively. Using that technical strategy, the positive loads of HHV-6A/-6B in her hair follicles were of 1.10 and 1.21 copies/cell, respectively (II:2 in Figure 2).

The inability of 14-day ganciclovir treatment to induce any clinical or virological effect on the HHV-6 viral load reinforced our hypothesis of iciHHV-6A+ and iciHHV-6B+. At Day 12, the HHV-6A and HHV-6B loads rose to 6.8 and 7.3 log_10_ copies/mL, respectively (Figure 1A). Without formal evidence of ongoing active viral replication, ganciclovir was stopped to avoid any undesirable and inappropriate antiviral treatment of our patient. The posttransplant longitudinal study, up to Day 170, confirmed high and stable levels of HHV-6A and -6B DNA in the blood, overall between 6.8–7.2 and 7.2–7.4 log_10_ copies/mL, respectively (Figure 1A). The patient remained asymptomatic under immunosuppressive treatment during this period.

To explore how both iciHHV-6A and iciHHV-6B were acquired, we analyzed hair follicle samples from all of the patient’s family members, including her father, mother, husband, and five children. All of them were healthy and immunocompetent, and none had a history of hepatitis or other infectious or inflammatory diseases. Except for her husband, whose testing was negative, her parents and all five children presented ~1 copy/cell of HHV-6A and/or HHV-6B detected in their hair follicles, as depicted in the pedigree chart of the family (Figure 2). More specifically, iciHHV-6A DNA was detected in the patient’s mother, and iciHHV-6B DNA was detected in her father. Our patient inherited both and transmitted the iciHHV-6A+ allele to three of her children, the iciHHV-6B+ allele to one child and both iciHHV-6A/6B alleles to the last child. This familial investigation reinforced the incidental finding of iciHHV-6A+ and iciHHV-6B+ in our patient and permitted us to demonstrate the Mendelian inheritance of distinct alleles carrying integrated viral sequences over three generations.

## 4. Discussion

We reported here a case of iciHHV-6A+ and iciHHV-6B+, incidentally discovered in the etiology of fulminant drug-induced hepatitis. iciHHV-6 individuals can be identified by their constant high DNAemia load over 5.5 or 6.0 log_10_ copies/mL of whole blood—according to techniques that are not yet standardized—usually corresponding to one copy per nucleated cell. Using quantitative PCR that detects HHV-6 and a reference cellular gene, the copy/cell ratio can be easily calculated for blood or extravascular samples such as hair follicles or nail clippings [12]. In addition to the lack of a clinical response, the persistence of high viral loads in blood under ganciclovir treatment combined with the detection of both HHV-6A and HHV-6B DNA in hair follicles permits us to conclude the latency of both iciHHV-6A and -6B variants.

Not all quantitative PCR assays differentiate HHV-6A and HHV-6B. Quantitative PCRs that distinguish HHV-6A and HHV-6B are currently recommended for the diagnosis of infection in patients with hematologic malignancies and after HSCT [17]. The multiplex quantitative PCR proved to be particularly useful in combined screening for both HHV-6 species, along with ganciclovir treatment and after SOT, *a fortiori*, in our rare iciHHV-6A+ and iciHHV-6B+ context. Aside from real-time PCR, droplet digital PCR has also been validated as a well-suited test to identify iciHHV-6A and -6B in cell and plasma samples [19].

Although HHV-6B has been proven to infect and induce direct cytopathic effects in human hepatocytes in vitro [20], acute liver failure due to HHV-6 remains very rare in immunocompetent adults [6,7,21]. Hepatitis is attributed to active HHV-6 infection/replication on the basis of: (i) elevated HHV-6 load in the blood and/or liver biopsy, (ii) significant decrease of the viral load and clinical response after ganciclovir administration, (iii) histological features of hepatocyte necrosis with virus-specific antigens immunoreactivity to early antigens, such as p41, or late structural proteins, such as glycoprotein H and VCA p140 of HHV-6B, or in situ hybridization or reverse transcription PCR for mRNA, all supporting an actively replicating viral process in hepatocytes, lymphocytes of portal inflammatory infiltrate or biliary epithelial tissue [22,23].

HHV-6 is an opportunistic pathogen in patients undergoing SOT or HSCT and reactivates in approximately 30–80% of patients approximately 2–6 weeks after transplantation. Most primary and HHV-6 reactivation associated with weak viral DNAemia are transient and asymptomatic in transplant patients. They otherwise can be responsible for complications, including graft rejection, acute graft-versus-host disease (stem cell), or multisystem organ dysfunction, resulting in fever, skin rash, myelosuppression, pneumonia or encephalitis [24]. HHV-6 has been weakly associated with hepatitis in SOT and HSCT [17]. In liver transplant patients, HHV-6 has been implicated as a cause of elevated transaminases, portal lymphocytic infiltration and confluent periportal necrosis that mimic acute cellular rejection [25]. The risk of HHV-6 severe infection and graft failure is increased in seronegative patients, most often children who develop a primary HHV-6 infection after transplantation, in patients showing evidence of HHV-6 active infection prior to the transplant, and in patients who do not receive ganciclovir prophylaxis against cytomegalovirus [24].

iciHHV-6 gives rise in healthy individuals to the production of higher amounts of U54 (a pp65 tegument protein)-specific CD8+ T cells [26] and higher levels of antibodies against viral immediate early (IE)1 U90, the product of *U90*, than do noniciHHV-6 individuals [27], reflecting an immune-controlled antigenic burden. The question of whether HHV-6 could fully reactivate from iciHHV-6 sequences was debated until subsequent phylogenetic analysis of replicative viruses and viral DNA isolated from hair follicles in a patient with X-linked severe combined immunodeficiency proved that iciHHV-6A can reactivate from its integrated state, produce virions and cause disease [28]. Moreover, it has been proven that, once integrated, HHV-6 can excise itself from human telomeres and reconstitute a full-length episome [29].

Environmental factors seem to affect the transcription of ciHHV-6A/B, which differ according to the specific human tissue. Aside from the highest levels of gene expression in the T-cells, brain, testis, esophagus, and adrenal gland, it has been demonstrated that ciHHV-6B is able to reactivate in hepatocytes as a permissive cell [27,30]. In immunosuppressed patients, reactivated HHV-6 from iciHHV-6 can induce acute liver failure and acute rejection, as demonstrated in a 5-year transplant recipient, by the presence of mRNA and late viral protein in the sole iciHHV-6B+ liver allograft [31]. Reactivated HHV-6 from iciHHV-6 is susceptible to antiviral drugs and can be treated with ganciclovir, valganciclovir, cidofovir, brincidofovir, or foscarnet [28,31,32,33,34]. However, antiviral treatment, considered a therapeutic option for iciHHV-6-associated diseases, leads to variable clinical improvement [24,33,35,36]. In our patient, ganciclovir did not improve liver function, and high levels of both HHV-6A and -6B DNAemia persisted at greater than 6.5 log_10_ copies/mL despite the initial antiviral treatment, not providing any evidence for reversible exogenous HHV-6 infection or active replication from iciHHV-6 as a primary cause of fulminant liver failure.

Although the pathomechanisms are complex and largely unknown, several observations illustrate a particular link between HHV-6 reactivation or antiviral immunity and adverse drug reactions in some individuals. Reactivation of HHV-6 with a high viral load in patients upon treatment with certain drugs, such as diverse antigout, antimicrobials, antiepileptics or anti-inflammatory medications, is a feature of drug-induced hypersensitivity syndrome (DRESS) or a drug-induced hypersensitivity reaction (DIHS) [37]. This highly variable syndrome is characterized by starting at least three weeks after first administration of the drug. It manifests with fever; lymphadenopathy; skin rash in 73 to 100% of the patients; hematologic abnormalities, eosinophilia being the most common in 66 to 95% of the patients; multisystem involvement, with hepatic involvement in 70% to 90% of the patients, who can progress to fulminant hepatic failure [38]; and viral reactivation mostly involving HHV-6 in 43% to 100% of the patients [39]. HHV-6 reactivation occurs opportunistically in susceptible individuals under conditions of transient immunosuppression, due to a direct toxic effect of the drug or its metabolites such as those transiently associated with the onset of DRESS. HHV-6 may drive, via a cytokine storm caused by either a drug-specific T-cell response or the development of a specific antiviral response in the infected tissue, the exacerbation of the lesions [40,41].

However, whether reactivation from iciHHV-6 could have worsened drug-induced liver injury due to ulipristal acetate [42], a selective progesterone receptor modulator used to treat uterine fibroids before 2020 and now suspended by the European Medicines Agency, remains elusive [43]. Indeed, cases of drug-induced hypersensitivity syndrome in iciHHV-6+ patients have rarely been reported [44,45]. It is known that *Chlamydia* infection and many drugs, such as histone deacetylase inhibitor-trichostatin A or suberoylanilide hydroxamic acid; carbamazepine; escitalopram oxalate; allopurinol; abacavir; and some hormones, such as hydrocortisone, oxytocin, and progesterone can be efficacious in activating ciHHV-6 in vitro. This results in upregulation of HHV-6 immediate early (IE) genes transactivation, such as *U79*, *U90*, and *U91* [46,47]. Reactivated HHV-6 from iciHHV-6 has biological consequences for the homeostasis of the immune system and may induce both cholesterol and fatty acid metabolism dysfunctions [48]. Indeed, IE/*U90* expression strongly inhibits type I interferon signaling in infected cells, which reinforces the initial immune evasion during reactivation [46,47]. Late viral proteins such as glycoprotein H/*U100* from iciHHV-6 may be deleterious by locally damaging multiple tissues [27] by boosting proinflammatory cytokines in immunosuppressed patients, as observed in HSCT acute graft-versus-host-disease contexts, in which HHV-6B reactivation from iciHHV-6 has been found to be an independent risk factor [49]. Finally, reactivated HHV-6 from iciHHV-6 produces several small noncoding (snc) viral RNAs able to alter multiple mitochondria-associated pathways, even in the absence of any detectable viral DNA replication or virionic production within the first days of activation [48,50]. In our case, due to the lack of knowledge, neither transcriptomic nor proteomic analysis of the liver was performed, and we did not have the ability to distinguish silent iciHHV-6A and/or -6B latent infection from transcription/fully reactivation of iciHHV-6A and/or -6B that may have contributed to the drug-induced liver injury [51].

Since no definitive conclusions can be drawn from a single case, continued follow-up and accumulation of cases of ciHHV-6 are necessary, as the natural history of iciHHV-6A individuals is still poorly understood. We described herein for the first time iciHHV-6A+ and -6B+ double integration, confirmed by the detection of ~1 copy per cell of both HHV-6A and HHV-6B DNA in hair follicles by specific multiplex quantitative PCR. As already reported both in vivo and in vitro, the HHV-6A and HHV-6B genomes can integrate into a chromosome in germinal cells, proposed as the ultimate mode for HHV-6 to achieve latency [11,52]. iciHHV-6+ individuals transmit integrated chromosomes to 50% of their descendants. Applying fluorescence in situ hybridization, Daibata et al. demonstrated the vertical transmission of iciHHV-6B DNA over three generations [53]. The same team reported in 1999 the transmission to a daughter of HHV-6 DNA carried by both chromosomes 22q13 and 1q44, identically to the site of viral integration of her mother and father, respectively, but without having determined whether the integrated species was HHV-6A and/or -6B [54]. Since then, several distinct integration sites, such as 6q, 7q22, 9q34.3, 10q26.3, 11p15.5, 17p13.3, 18p11.3, 18q23, 19q13.4, and Xp, have been described in telomeric regions [55]. Herein, we report that iciHHV-6A and iciHHV-6B can coexist, and the presence of HHV-6A and/or -6B DNA in hair follicles was demonstrated over three generations in the same family.

## 5. Conclusions

In the actual context of increasing risks of HHV-6A and HHV-6B coinfection [56] and the prevalence suggesting that nearly 70 million individuals worldwide carry iciIHHV-6 [16,57,58], our case report highlights the need for better screening of iciHHV-6A/-6B+ patients. Distinguishing iciHHV-6A and -6B more commonly in routine diagnosis would allow better detection and may elicit further studies on the biological and immunological specificities of the two integrated species. In this respect, duplex HHV-6A/6B real-time quantitative PCR allows rapid investigation of whole blood and hair follicles, which allowed us to engage in rapid decision making in the context of drug-induced fulminant hepatitis and liver transplantation. Furthermore, although no biomarkers are currently available to identify susceptible patients, iciHHV-6A/-6B screening could be proposed before the administration of drugs known to be associated with virus-mediated adverse reactions to identify patients more at greater risk of HHV-6 reactivation and severe outcomes. Given the rarity of this presentation, it can only be assumed that cointegration of iciHHV-6A and iciHHV-6B may have played a role in the development or severity of our patient’s idiosyncratic drug-induced liver injury; however, future studies are needed to determine whether the presence of both iciHHV-6A+ and iciHHV-6B+ constitutes a higher risk factor for the development of any inflammatory or malignant diseases [16].

## Data Availability

Not applicable.
